# Analysis of chemotherapy-induced peripheral neuropathy using the Japanese Adverse Drug Event Report database

**DOI:** 10.1038/s41598-021-90848-6

**Published:** 2021-05-31

**Authors:** Misaki Inoue, Kiyoka Matsumoto, Mizuki Tanaka, Yu Yoshida, Riko Satake, Fumiya Goto, Kazuyo Shimada, Ririka Mukai, Shiori Hasegawa, Takaaki Suzuki, Hiroaki Ikesue, Jun Liao, Tohru Hashida, Mitsuhiro Nakamura

**Affiliations:** 1 Laboratory of Drug Informatics, Gifu Pharmaceutical University, Gifu-shi, Gifu Japan; 2 Department of Pharmacy, Kobe City Medical Center General Hospital, Kobe-shi, Hyogo Japan; 3 Gifu Prefectural Government, Gifu-shi, Gifu Japan; 4 Department of Pharmaceutical Informatics and Biological Statistics, School of Science, China Pharmaceutical University, Nanjing, China

**Keywords:** Adverse effects, Chemotherapy

## Abstract

Chemotherapy-induced peripheral neuropathy (CIPN) is a common adverse event associated with several antineoplastic drugs; however, the precise risks and time course of reactions of particular drugs are not clearly understood. The aim of this study was to evaluate the relationship between anticancer agents and CIPN development using data from the Japanese Adverse Drug Event Report (JADER) database and to characterize the time-to-onset and outcomes of CIPN. Chemotherapy-induced peripheral neuropathy was defined using the Medical Dictionary for Regulatory Activities preferred terms. Disproportionality analysis was performed by calculating the reporting odds ratio (ROR) with 95% confidence interval for signal detection. Data of nine Anatomical Therapeutic Chemical (ATC) drug categories correlated with CIPN development, in addition to the data of the time-to-onset and outcomes. Among 622,289 reports in the JADER database from April 2004 to March 2020, there were 1883 reports of adverse events corresponding to peripheral neuropathy. The ROR (95% confidence interval) for vinblastine, sorbent-based paclitaxel (sb-PTX), oxaliplatin, and bortezomib was 20.4 (12.5–33.4), 13.6 (11.9–15.7), 26.2 (23.6–29.1), and 30.8 (26.6–35.8), respectively. The median duration (interquartile range) to CIPN development after the administration of vinca alkaloids and analogues, taxanes, platinum compounds, and monoclonal antibodies was 11.0 (5.0–46.5), 22.5 (6.0–82.5), 22.0 (6.0–68.5), and 32.5 (11.3–73.8) days, respectively. The median duration (interquartile range) of sb-PTX and nanoparticle albumin-bound (nab)-PTX was 35.0 (7.0–94.0) and 5.5 (3.0–29.3) days, respectively. Our analysis of records in the JADER database revealed several drugs associated with a high risk for CIPN development. In particular, the development of CIPN after vinca alkaloid administration should be closely monitored for 2 weeks after administration. CIPN caused by nab-PTX showed significantly faster onset than that by sb-PTX. Patients who receive taxanes or monoclonal antibodies often do not show an improvement; accordingly, early treatment is required.

## Introduction

Chemotherapy-induced peripheral neuropathy (CIPN) is a common adverse event (AE) associated with several antineoplastic drugs^1,2^. It usually presents as a typical “glove and stocking” neuropathy and is commonly characterized by numbness, tingling, and neuropathic pain in the extremities. Furthermore, long sensory nerves are susceptible, and autonomic and motor dysfunctions might occur^3–6^. Antineoplastic drugs that frequently cause CIPN are platinum compounds (e.g., carboplatin, cisplatin, and oxaliplatin), taxanes (e.g., paclitaxel (PTX) and docetaxel), epothilones (e.g., ixabepilone), vinca alkaloids (e.g., vincristine and vinblastine), bortezomib, and thalidomide^1,2,4,7,8^. Chemotherapy-induced peripheral neuropathy is a major dose-limiting adverse effect of several first-line chemotherapeutic agents^3^. The development of CIPN may result in chemotherapy dose reduction or cessation^9^; it can lead to long-term debilitating effects, with increased morbidity and decreased quality of life^10^.

A systematic review reported that 1960 patients developed CIPN (aggregate prevalence 48%). Furthermore, the prevalence of CIPN was 68.1% within the first month of the end of chemotherapy, 60% at 3 months, and 30% at 6 months or more^9^. Drug-specific manifestations, such as acute neurotoxicity of PTX and oxaliplatin^2,8^, and exacerbation of symptoms after the discontinuation of cisplatin (coasting)^2,8,11^ may occur. Neuropathy induced by taxanes and bortezomib is usually reversible and improves or resolves within a few months after the discontinuation of treatment; however, chronic symptoms induced by platinum-based drugs and thalidomide may persist^8,12^. Despite extensive research on CIPN, information about the time-to-onset and outcomes for each drug is limited. Thus, there is a need for strategies for CIPN prevention and treatment^1^. Information about time-to-onset and outcome of CIPN is considered valuable for early monitoring of AEs by healthcare professionals in the field of oncology.

Paclitaxel has been formulated and marketed as solvent-based (sb)-PTX (Taxol^®^; Bristol-Myers Squibb, New York, NY, USA) since 1992. Solvent-based PTX contains a combination of Cremophor EL (a synthetic, nonionic surfactant) and ethanol (co-solvent) as an excipient. Solvent-free, nanoparticle albumin-bound PTX (nab-PTX) (Abraxane^*®*^; Celgene Corporation, Summit, NJ, USA) was developed in 2005. It is characterized by rapid and preferential delivery as well as accumulation of PTX at tumor sites, thereby enhancing the therapeutic effects of PTX^13–15^. Furthermore, nab-PTX displays a reasonable toxicity profile, avoiding solvent/surfactant-related AEs such as hypersensitivity reactions and the need for premedication^13,16^. However, the detailed AE profile of PTX formulations in clinical setting is uncertain.

The regulatory authority in Japan, the Pharmaceuticals and Medical Devices Agency (PMDA), controls spontaneous reporting systems (SRSs) of the Japanese Adverse Drug Event Report (JADER) database. Spontaneous reporting systems serve as a valuable tool for post-marketing surveillance, reflecting the realities of clinical practice. There are numerous well-controlled clinical studies; however, we believe that it is important to understand the occurrence of AEs under complex patient backgrounds and drug treatments in clinical practice. In this study, we analyzed data from the JADER database to comprehensively evaluate CIPN development. Analyses of CIPN development using data from the SRS database are rare; to the best of our knowledge, this is the first study on the relationship between anticancer agents and peripheral neuropathy by outcome and time-to-onset analyses. Furthermore, we assessed the AE profiles of sb-PTX and nab-PTX formulations.

## Results

**Figure 1:**
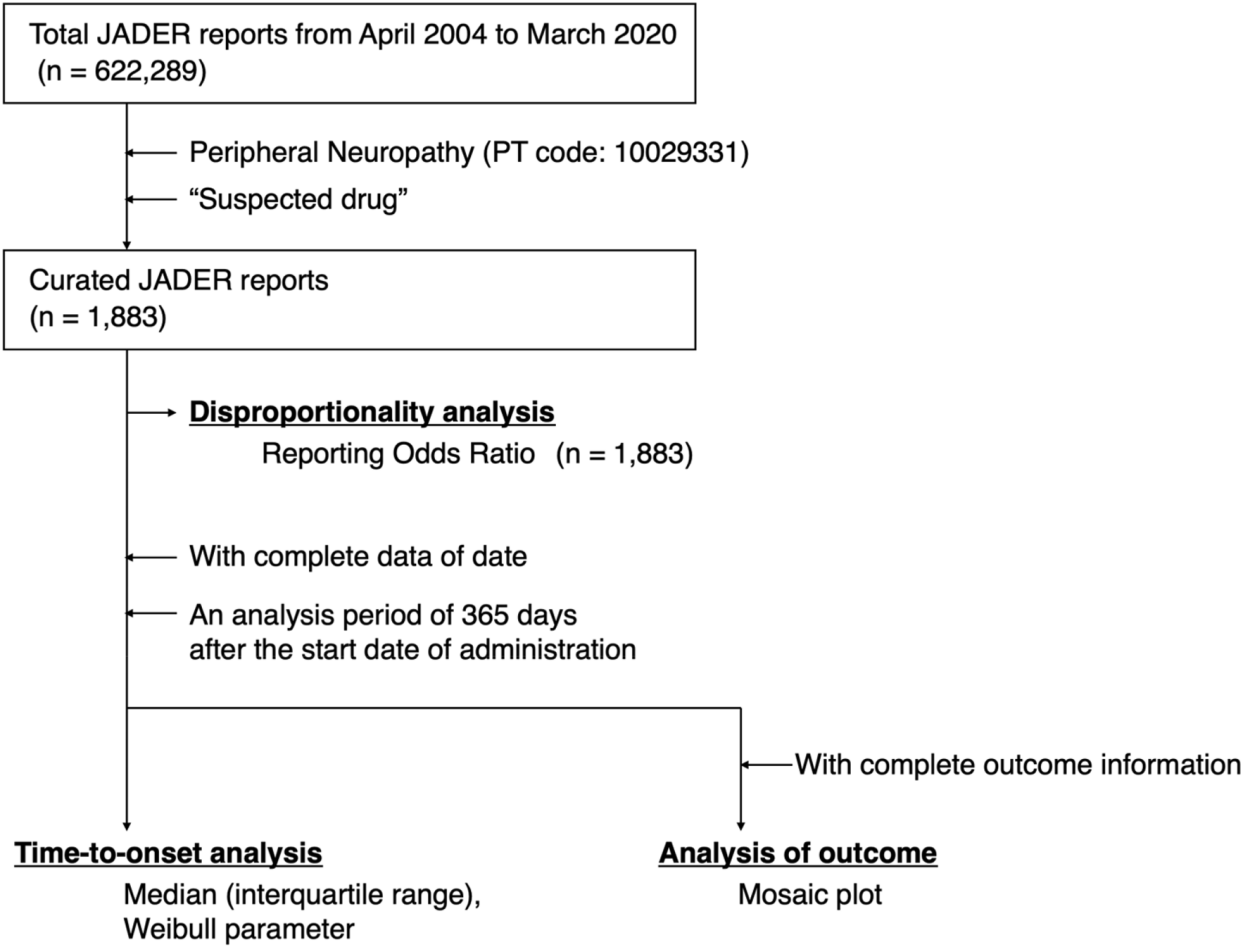
Flowchart outlining the construction of the dataset used for analysis.

The JADER database contained 622,289 reports from April 2004 to March 2020. The number of AE reports corresponding to peripheral neuropathy was 1,883 (Fig. 1). Drugs with more than 20 records in the database and antitumor drugs with previously reported associations with neuropathy^1,2,4,7,8^ are summarized in Table 1. Although the number of reports was 20 or less, “purine analogues” and “protein kinase inhibitors” were added to Table 1 because the time-to-onset analysis was possible. For the purposes of this study, data from the JADER database listed as “paclitaxel protein-bound particles for injectable suspension” and “paclitaxel” were classified as nab-PTX and sb-PTX, respectively. The 10 most frequently reported drugs were oxaliplatin (491 cases), sb-PTX (231 cases), bortezomib (210 cases), bevacizumab (121 cases), fluorouracil (116 cases), carboplatin (87 cases), lenalidomide (70 cases), nivolumab (54 cases), vincristine (49 cases), and capecitabine (46 cases). The drugs for which the lower limit of the 95% confidence interval (CI) of reporting odds ratio (ROR)^17,18^ exceeded 1 and ROR exceeded 10 were as follows: nelarabine, vinblastine, sb-PTX, oxaliplatin, brentuximab vedotin, daratumumab, cediranib, lorlatinib, bortezomib, and thalidomide. Their RORs (95% CIs) were 37.2 (22.5–61.5), 20.4 (12.5–33.4), 13.6 (11.9–15.7), 26.2 (23.6–29.1), 22.0 (15.9–30.4), 14.3 (10.0–20.4), 14.8 (5.4–40.4), 30.1 (15.2–59.6), 30.8 (26.6–35.8), and 12.4 (7.1–21.5), respectively.

**Table 1:** Number of reports and reporting odds ratios for chemotherapy-induced peripheral neuropathy.

Category	ATC^a^ code	Drugs	Total (n)	Case (n)	ROR^b^ (95% CI^c^)
Total			622,289	1883	
Purine analogues^f^	L01BB05	fludarabine	1498	3	0.7 (0.2–2.1)
	L01BB07	nelarabine	169	17	37.2 (22.5–61.5)*
Pyrimidine analogues	L01BC02	fluorouracil	8678	116	4.7 (3.9–5.7) *
	L01BC05	Gemcitabine	4939	29	2.0 (1.4–2.8) *
	L01BC06	Capecitabine	4022	46	3.9 (2.9–5.2) *
Vinca alkaloids and analogues	L01CA01	Vinblastine	294	17	20.4 (12.5–33.4) *
	L01CA02	Vincristine	3293	49	5.1 (3.8–6.8) *
	L01CA03	Vindesine	227	2	2.9 (0.7–11.8)
	L01CA04	Vinorelbine	800	4	1.7 (0.6–4.4)
Taxanes	L01CD01	Sorbent-based paclitaxel	6529	231	13.6 (11.9–15.7) *
	L01CD01	Nanoparticle albumin-bound paclitaxel	1673	27	5.5 (3.7–8.0) *
	L01CD02	Docetaxel	7274	40	1.8 (1.3–2.5) *
	L01CD04	Cabazitaxel	1365	9	2.2 (1.1–4.2) *
Anthracyclines and related substances	L01DB01	Doxorubicin	4540	24	1.8 (1.2–2.6) *
Platinum compounds	L01XA01	Cisplatin	9488	32	1.1 (0.8–1.6)
	L01XA02	Carboplatin	6212	87	4.9 (3.9–6.0) *
	L01XA03	Oxaliplatin	8737	491	26.2 (23.6–29.1) *
	–^d^	Miriplatin hydrate	312	1	–^e^
	–^d^	Nedaplatin	581	1	–^e^
Monoclonal antibodies	L01XC07	Bevacizumab	11,048	121	3.8 (3.2–4.6) *
	L01XC12	Brentuximab vedotin	652	40	22.0 (15.9–30.4) *
	L01XC17	Nivolumab	8099	54	2.2 (1.7–2.9) *
	L01XC18	Pembrolizumab	4928	35	2.4 (1.7–3.3) *
	L01XC24	Daratumumab	782	32	14.3 (10.0–20.4) *
Protein kinase inhibitors^f^	L01XE01	Imatinib	4747	6	0.4 (0.2–0.9)
	L01XE02	Gefitinib	2863	1	–^e^
	L01XE04	Sunitinib	3631	3	0.3 (0.1–0.8)
	L01XE05	Sorafenib	5261	5	0.3 (0.1–0.7)
	L01XE06	Dasatinib	1534	3	0.6 (0.2–2.0)
	L01XE07	Lapatinib	753	3	1.3 (0.4–4.1)
	L01XE08	Nilotinib	2056	1	–^e^
	L01XE10	Everolimus	4070	3	0.2 (0.1–0.8)
	L01XE11	Pazopanib	1712	2	0.4 (0.1–1.5)
	L01XE14	Bosutinib	390	1	–^e^
	L01XE16	Crizotinib	1180	6	1.7 (0.8–3.8)
	L01XE17	Axitinib	1052	2	0.6 (0.2–2.5)
	L01XE18	Ruxolitinib	1172	3	0.8 (0.3–2.6)
	L01XE21	Regorafenib	1957	6	1.0 (0.5–2.3)
	L01XE23	Dabrafenib	312	1	–^e^
	L01XE24	Ponatinib	426	3	2.3 (0.8–7.3)
	L01XE25	Trametinib	321	1	–^e^
	L01XE27	Ibrutinib	396	1	–^e^
	L01XE32	Cediranib	93	4	14.8 (5.4–40.4) *
	L01XE33	Palbociclib	1572	1	–^e^
	L01XE44	Lorlatinib	108	9	30.1 (15.2–59.6) *
	L01XE54	Gilteritinib	194	1	–^e^
Other antineoplastic agents	L01XX19	Irinotecan	6136	38	2.1 (1.5–2.9) *
	L01XX32	Bortezomib	2727	210	30.8 (26.6–35.8) *
	L01XX50	Ixazomib	893	21	8.0 (5.2–12.4) *
Other immunosuppressants	L04AX02	Thalidomide	362	13	12.4 (7.1–21.5) *
	L04AX04	Lenalidomide	5714	70	4.2 (3.3–5.3)*
	L04AX06	Pomalidomide	1378	3	0.7 (0.2–2.2)

^a^ATC: Anatomical Therapeutic Chemical.

^b^ROR: Reporting Odds Ratio.

^c^CI: Confidence Interval.

^d^ATC code has not been assigned.

^e^Number of cases was < 2.

^f^Although the number of reports was 20 or less, “purine analogues” and “protein kinase inhibitors” were added because the time-to-onset analysis was possible.

*Lower limit of the 95% CI corresponding to the ROR was greater than 1.

**Figure 2:**
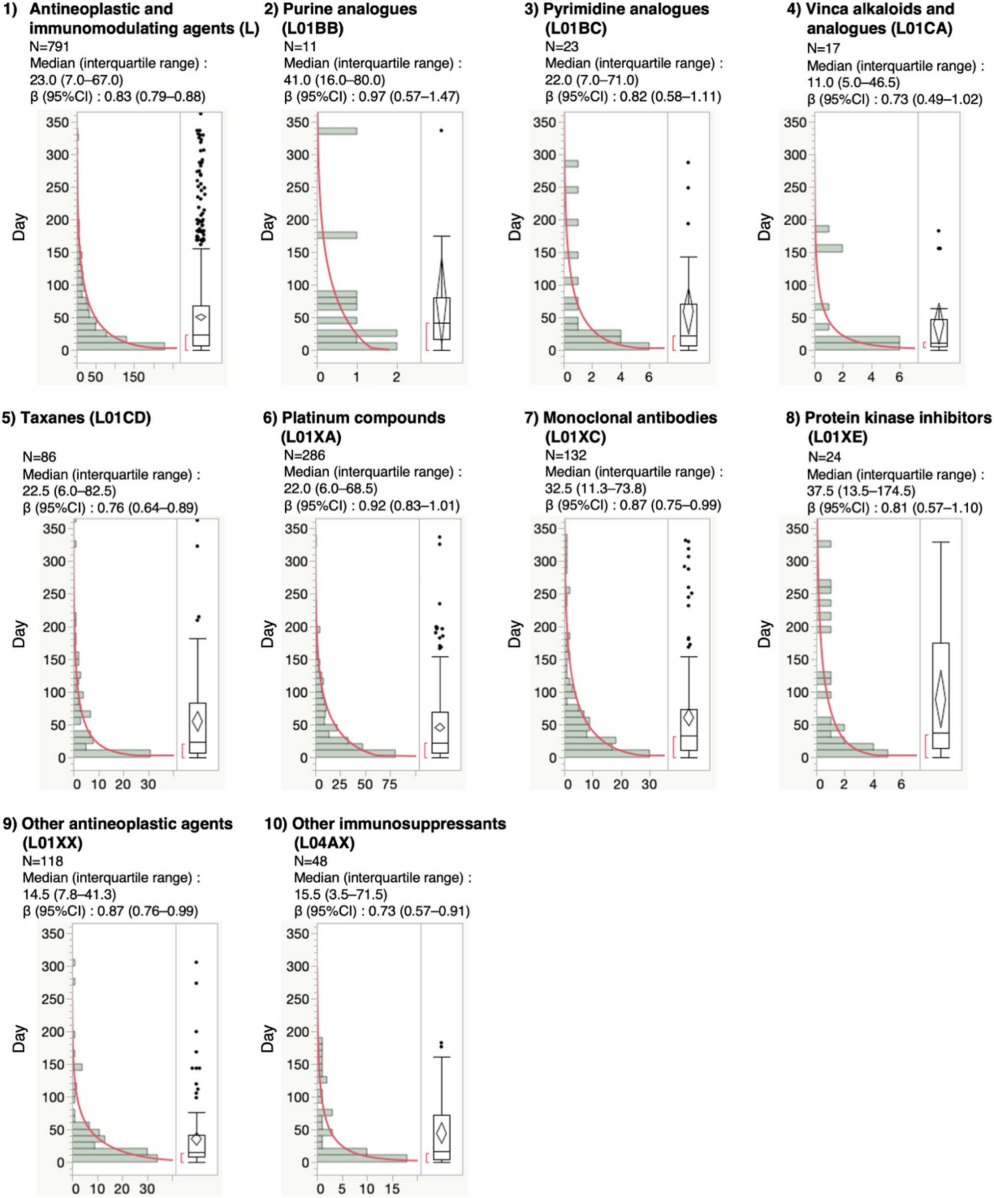
Histogram and Weibull shape parameter of chemotherapy-induced peripheral neuropathy for each drug in the ATC classification. Right panel shows box plots, which represent the median (the horizontal line within the box). The ends of the box represent the 25th and 75th quantiles, also expressed as the 1st and 3rd quartile, respectively. The confidence diamond contains the mean and the upper and lower 95% CIs of the mean. The whiskers extend to the outermost data point that falls within the distances of 1.5 times the length of the inner quartiles. The bracket outside the box indicates the shortest half, which is the densest 50% of the observations.

**Figure 3:**
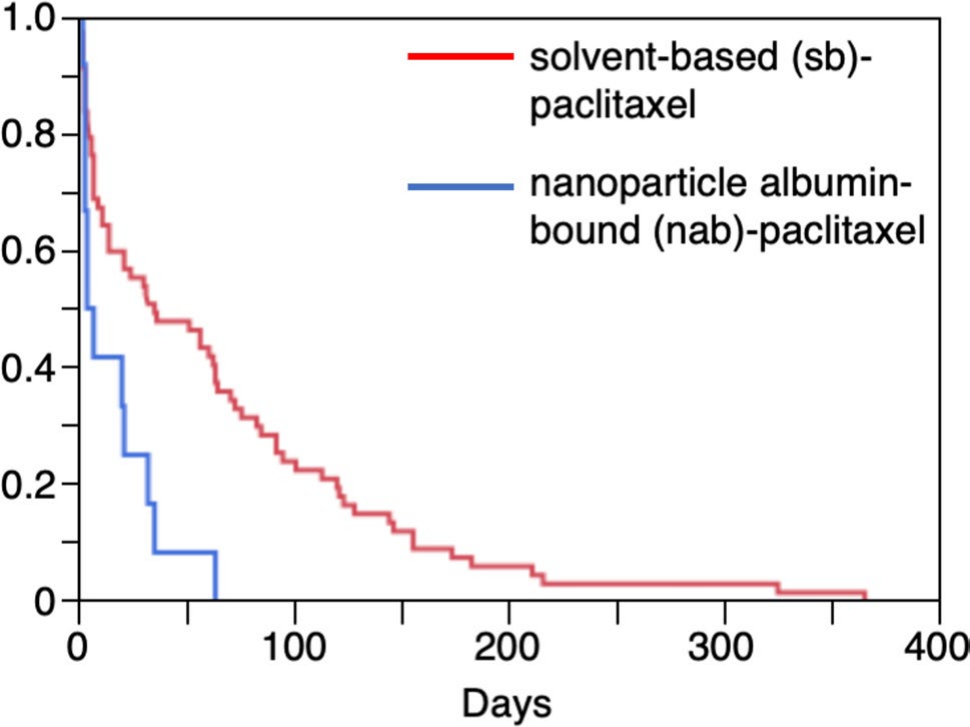
Kaplan–Meier plot of chemotherapy-induced peripheral neuropathy for solvent-based (sb)-paclitaxel and nanoparticle albumin-bound (nab)-paclitaxel.

For the time-to-onset analysis, we extracted combinations with complete information for the date of treatment initiation and date of AE onset. We evaluated nine Anatomical Therapeutic Chemical (ATC) drug classes with more than 10 reported cases (Table 2 and Fig. 2). The median duration (interquartile range) for CIPN development due to the administration of purine analogues (ATC code: L01BB), pyrimidine analogues (ATC code: L01BC), vinca alkaloids and analogues (ATC code: L01CA), taxanes (ATC code: L01CD), platinum compounds (ATC code: L01XA), monoclonal antibodies (ATC code: L01XC), protein kinase inhibitors (ATC code: L01XE), other antineoplastic agents (ATC code: L01XX), and other immunosuppressants (ATC code: L04AX) was 41.0 (16.0–80.0), 22.0 (7.0–71.0), 11.0 (5.0–46.5), 22.5 (6.0–82.5), 22.0 (6.0–68.5), 32.5 (11.3–73.8), 37.5 (13.5–174.5), 14.5 (7.8–41.3), and 15.5 (3.5–71.5) days, respectively. The upper limit of the 95% CI of the Weibull shape parameters (WSP) β-value^19^ for taxanes, monoclonal antibodies, other antineoplastic agents, and other immunosuppressants was less than 1. The median duration (interquartile range) of sb-PTX (n = 67) and nab-PTX (n = 12) administration was 35.0 (7.0–94.0) and 5.5 (3.0–29.3) days, respectively. In addition, CIPN by nab-PTX showed significantly faster onset than that by sb-PTX (*P* = 0.002) (Table 2 and Fig. 3).

**Table 2:** The medians and Weibull parameter of chemotherapy-induced peripheral neuropathy.

ATC classification (ATC code)	Total (ROR)	Case for ROR calculation (n)	Case for time-to-onset analysis (n)	Median (interquartile range)	Scale parameter, α (95% CI)	Shape parameter, β (95% CI)
*Antineoplastic and immunomodulating agents (L)*	238,138	2222	791	23.0 (7.0–67.0)	49.09 (44.74–53.80)	0.83 (0.79–0.88)
*Purine analogues (L01BB)*	1667	20	11	41.0 (16.0–80.0)	82.73 (38.45–169.76)	0.97 (0.57–1.47)
*Pyrimidine analogues (L01BC)*	28,960	212	23	22.0 (7.0–71.0)	58.29 (32.19–102.05)	0.82 (0.58–1.11)
*Vinca alkaloids and analogues (L01CA)*	4387	70	17	11.0 (5.0–46.5)	33.95 (15.61–70.42)	0.73 (0.49–1.02)
*Taxanes (L01CD)*	18,506	333	86	22.5 (6.0–82.5)	46.88 (34.58–62.87)	0.76 (0.64–0.89)
sorbent-based paclitaxel (L01CD01)	6529	231	67	35.0 (7.0–94.0)	56.24 (40.06–77.91)	0.78 (0.64–0.94)
nanoparticle albumin-bound paclitaxel (L01CD01)	1673	27	12	5.5 (3.0–29.3)	13.01 (4.93–32.05)	0.74 (0.44–1.13)
*Platinum compounds (L01XA)*	24,749	611	286	22.0 (6.0–68.5)	50.22 (43.48–57.83)	0.92 (0.83–1.01)
carboplatin (L01XA02)	6212	87	20	32.0 (3.8–83.8)	49.54 (26.34–89.70)	0.87 (0.57–1.26)
oxaliplatin (L01XA03)	8737	491	259	22.0 (7.0–68.0)	51.48 (44.40–59.52)	0.95 (0.85–1.05)
*Monoclonal antibodies (L01XC)*	44,931	374	132	32.5 (11.3–73.8)	60.22 (48.38–74.53)	0.87 (0.75–0.99)
bevacizumab (L01XC07)	11,048	121	48	15.0 (7.0–56.0)	50.09 (32.25–76.35)	0.78 (0.61–0.97)
*Protein kinase inhibitors (L01XE)*	21,336	50	24	37.5 (13.5–174.5)	82.90 (46.74–142.25)	0.81 (0.57–1.10)
*Other antineoplastic agents (L01XX)*	12,835	320	118	14.5 (7.8–41.3)	33.19 (26.45–41.41)	0.87 (0.76–0.99)
bortezomib (L01XX32)	2727	210	80	17.5 (8.5–40.0)	31.80 (24.57–40.83)	0.94 (0.79–1.10)
*Other immunosuppressants (L04AX)*	7454	86	48	15.5 (3.5–71.5)	39.20 (25.15–59.86)	0.73 (0.57–0.91)
lenalidomide (L04AX04)	5714	70	35	13.0 (2.0–43.0)	26.80 (15.13–46.16)	0.66 (0.50–0.84)

**Figure 4:**
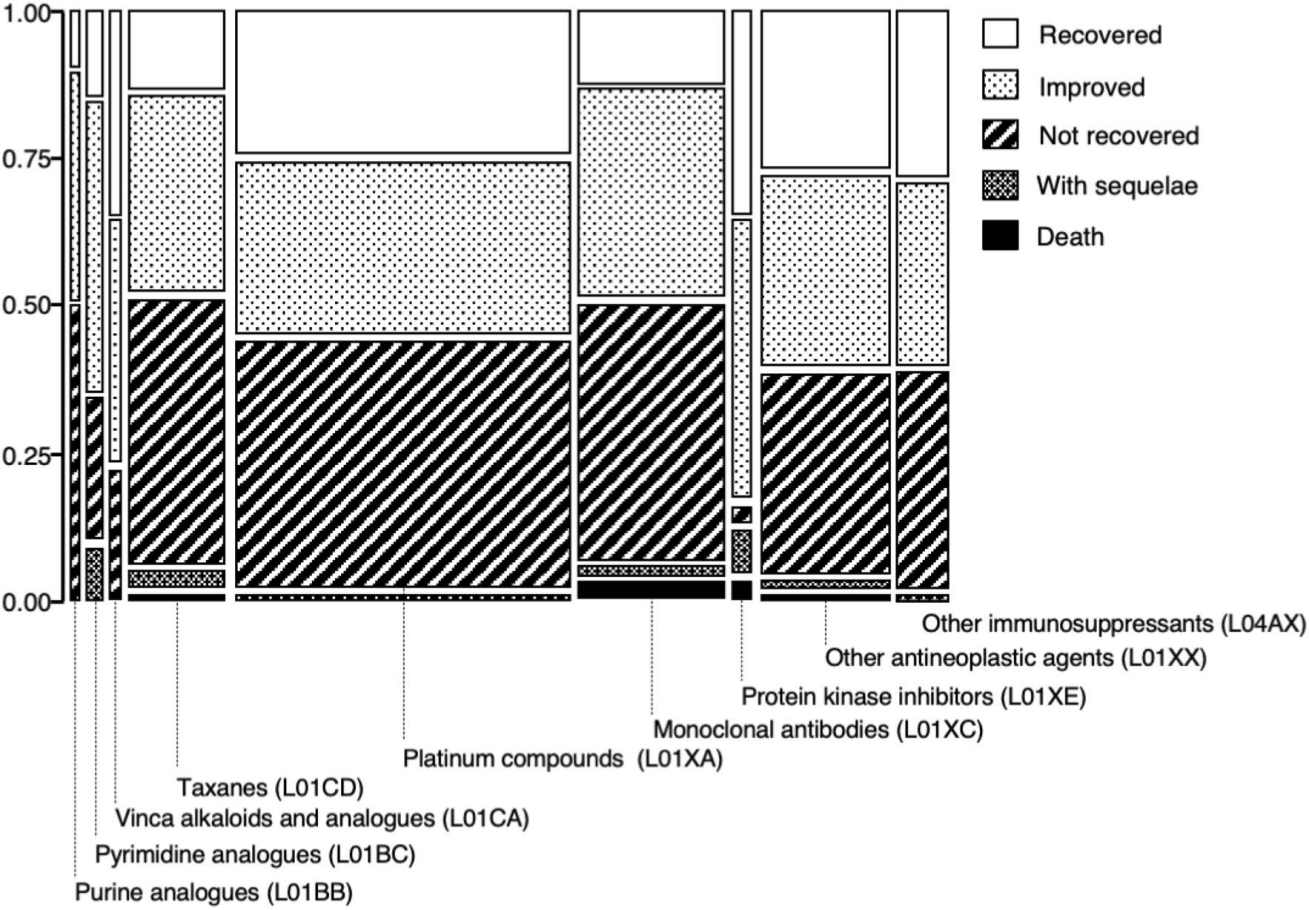
Mosaic plot of outcomes of chemotherapy-induced peripheral neuropathy. The plot is divided into rectangles where each vertical length represents the proportion of each level of the Y variable within each level of the X variable.

We generated a mosaic plot to summarize the outcome profiles for CIPN stratified by the nine ATC classes, as shown in Fig. 4. The rates of “recovered” or “improved” outcomes were greater than 50% for the ATC classes, except for purine analogues, taxanes and monoclonal antibodies, with rates of 76.5% (13/17 cases) for vinca alkaloids and analogues and 82.6% (19/23 cases) for protein kinase inhibitors. The combined frequency of “death,” “with sequelae,” and “not recovered” was greater than 40% for purine analogues, taxanes, platinum compounds, and monoclonal antibodies, with the highest frequency [i.e., 51.8% (44/85 cases)] for taxanes. The most frequent outcome for the total cases was “not recovered” [39.7% (285/717 cases)]s and the frequency of “death” was 1.7%. The combined frequency of “death (2 cases),” “with sequelae (2 cases),” and “not recovered (34 cases)” associated with sb-PTX was 56.7% (38/67 cases). The combined frequency of “death (0 case),” “with sequelae (0 case),” and “not recovered (4 cases)” associated with nab-PTX was 33.3% (4/12 cases).

As multiple drugs are included in one ATC unit, it is difficult to accurately evaluate the time-to-onset profile and outcome profile of individual drugs. We examined the drugs that had over 50 reports (Table 1). The median duration (interquartile range) of CIPN caused by carboplatin (n = 20), oxaliplatin (n = 259), bevacizumab (n = 48), bortezomib (n = 80), and lenalidomide (n = 35) was 32.0 (3.8–83.8), 22.0 (7.0–68.0), 15.0 (7.0–56.0), 17.5 (8.5–40.0), and 13.0 (2.0–43.0) days, respectively. The upper limit of the 95% CI of the WSP β-value for sb-PTX, bevacizumab, and lenalidomide was less than 1 (Table 2). The combined rate of “recovered” or “improved” outcome for carboplatin, oxaliplatin, bevacizumab, bortezomib, and lenalidomide was 77.8% (14/18 cases), 54.5% (139/255 cases), 46.8% (22/47 cases), 58.7% (44/75 cases), and 63.6% (21/33 cases), respectively. The combined frequency of “death,” “with sequelae,” and “not recovered” was 22.2% (4/18 cases), 45.5% (116/255 cases), 53.2% (25/47 cases), 41.3% (31/75 cases), and 36.4% (12/33 cases), respectively.

## Discussion

We detected AE signals for CIPN caused by several drugs in the JADER database. The risk of CIPN development due to the administration of platinum compounds, taxanes, vinca alkaloids, bortezomib, and thalidomide has been reported^1,2,4,7,8^, consistent with our results. As Cremophor EL is associated with peripheral neuropathies probably due to axonal degeneration^20–23^, sb-PTX might cause neuropathy. It has been reported that nab-PTX is associated with a high risk of CIPN development due to a high single dose (260 mg/m^2^)^24^. In a previous study using data from the US Food and Drug Administration (FDA) Adverse Event Reporting System (FAERS) database, no difference was observed in the ROR for CIPN between nab-PTX and sb-PTX^25^. In this study, which used data from the JADER database, the ROR of sb-PTX was higher than that of nab-PTX. We do not have a conclusive explanation for our finding. Platinum compounds, taxanes, vinca alkaloids, and bortezomib are used in various chemotherapeutic regimens such as the fluorouracil plus oxaliplatin (FOLFOX) regimen. The FOLFOX regiment plus bevacizumab is commonly administered to patients with metastatic colorectal cancer^26^. The ROR signals for pyrimidine analogues, doxorubicin, and bevacizumab are generally not associated with CIPN development^12^. The ROR signals for pyrimidine analogues, doxorubicin, and bevacizumab detected in our study can likely be explained by anticancer agents (e.g., those involving platinum compounds) used in combination. Owing to the limitations of SRSs, disproportionality measures, such as RORs, are often used to detect statistical associations based on signal strength; however, these measures do not quantify risk or demonstrate causality^17,18^. The ROR is an indicator of an increased risk of AE reporting, but it does not indicate the risk of AE occurrence in absolute terms^17,18^; therefore, the results of this study should be interpreted with caution.

Brentuximab vedotin induces CIPN after long-term exposure and is mostly reversible^27^. Immune checkpoint inhibitors (e.g., nivolumab and pembrolizumab) have the potential to cause adverse reactions in the nervous system, either acutely or subacutely. Immune checkpoint inhibitor-induced peripheral neuropathy reportedly occurs in less than 1% of patients and is a rare complication^28^. As it is a relatively new drug, further research is needed.

We found that more than 50% of CIPN cases associated with pyrimidine analogues, vinca alkaloids and analogues, taxanes, platinum compounds, other antineoplastic agents, and other immunosuppressants occurred within 4 weeks. Vinca alkaloids and analogues had the shortest median time from drug administration to CIPN onset, that is, within 14 days, among the nine ATC classes. The upper limit of the 95% CI of the WSP β-value for taxanes, monoclonal antibodies, other antineoplastic agents, and other immunosuppressants was less than 1, and the hazard decreased over time (initial failure type).

Here, nab-PTX (median 5.5 days) was associated with a significantly faster onset of CIPN than sb-PTX (median 35.0 days). This finding based on actual clinical data is considered important for clinicians. nab-PTX has several differences in the formulation parameters (a larger volume of distribution, larger clearance, higher fraction of unbound drug, higher systemic exposure, and maximal concentration of unbound drug relative to sb-PTX)^29,30^. The early onset of CIPN by nab-PTX may be explained by the differences in these parameters and the high doses.

The symptoms of vincristine-induced peripheral neuropathy often develop after a few doses, and in most cases, disappear a few months after the discontinuation of vincristine^31^. Death and sequelae related to vincristine were not reported in the JADER database.

Cisplatin and oxaliplatin cause coasting^2,8,11^, in which symptoms persist for a long period, even after discontinuation, and oxaliplatin is associated with acute and chronic symptoms that occur immediately after administration^11^. The pathogenesis of CIPN is not completely understood; however, several mechanisms have been proposed. Neurotoxic effects are triggered by drug accumulation in the dorsal root ganglia, causing neuronal dysfunction and apoptosis, axonal degeneration due to microtubule inhibition, mitochondrial dysfunction, inflammation, and oxidative stress^2,3,7,12,32^, leading to long-term, often irreversible, changes in the peripheral nervous system^11,33,34^. Metabolites of oxaliplatin, such as oxalate, may cause the extension of the opening of voltage-gated Na^+^ channels, overexciting peripheral nerves, and induce acute peripheral neuropathy^4,10^. Variations in the time-to-onset and severity of adverse effects may be due to the differences in the underlying mechanism. In this study, we did not directly focus on pharmacological findings. Attempts have been made in the area of drug repositioning or translational research to search for genes related to AEs inspired by simple ROR values^35,36^. Expansion into such areas in the future will be a challenge.

Our study had some limitations that should be noted. The SRSs are subject to over-reporting, under-reporting, missing data, the exclusion of healthy individuals, a lack of denominators, and confounding factors. The ROR does not provide sufficient evidence for causality and only offers a rough indication of signal strength. To date, no method has been widely accepted for adjusting covariates in studies using SRS datasets. Multiple logistic analysis may be an approach to deal with covariates that affect the reliability of the results^37,38^. The use of propensity scores^39^ to reduce bias by equating groups based on possible covariates or other appropriate parameters^40–42^ would be a useful approach. In the future, we will attempt to adjust these biases. As the JADER database is a collection of voluntary reports, it may not contain reports of the mildest grade of CIPN, resulting in underestimation of the prevalence of CIPN. The JADER database does not contain detailed information, such as clinical background (e.g., diabetes)^3,12^, types and stages of cancers, and chemotherapy regimens. Several anticancer drugs are often used in combination, but drug combinations were not considered in this analysis. The time-to-onset/outcome analysis was performed for ATC units. As reports with incomplete data of date or outcome were excluded from the analysis, we did not analyze drugs with fewer reports (Table 2). It is worthwhile to analyze after a sufficient number of cases have been accumulated, but that is a topic for future research. Further epidemiological studies might be required to confirm these results. These issues must be fully considered when analyzing drug safety using SRS data.

Our results are derived from real-world datasets, such as the JADER database, that are affected by patient backgrounds and concomitant medications, which are not extensively discussed in clinical studies. In spontaneous report analysis, easy risk assessment using the disproportionality analysis such as the ROR index should be refrained. It is clear that a controlled intervention trial would provide an accurate risk assessment. On the contrary, it has been reported that none of the methods (e.g., clinical trials and cohort studies) if taken alone should be considered definitive for evaluating drug risk, and disproportionality studies are therefore important^43^. Furthermore, clinical research focusing on AEs might be generally unattractive to stakeholders who actively promote clinical research, and there are only a few actual cases. Clinically useful findings from simple time-to-onset and ROR analysis results have already been reported^44–46^. To evaluate the relationship between drug administration and AEs, it is useful to evaluate the results of disproportionality analysis based on frequency information and time-to-onset that can evaluate the changes in the risk of AEs over time. Although our research results are not comprehensive, we consider them valuable because they are complementary to the results of existing clinical studies. If oncologists and general practitioners know the timing and outcome profiles such as ROR, time-to-onset, and outcome of CIPN that actually occurs in clinical practice based on real-world data, early intervention would be possible, and this can reduce the risk of overlooking CIPN. Our study indicates the importance of comparing the safety profiles using post-marketing real-world data.

## Conclusions

Despite the limitations inherent to SRSs, our results confirmed the CIPN risk for various agents based on RORs and time‐to‐onset analyses. CIPN was more likely to develop at an early time point after drug administration, emphasizing the importance of careful monitoring, especially during the first 2 months. Patients treated with vinca alkaloids, such as vincristine, often develop CIPN within 2 weeks and show subsequent recovery or improvement. CIPN caused by nab-PTX showed significantly faster onset than that by sb-PTX. Patients who receive taxanes or monoclonal antibodies often do not show an improvement and early treatment is required.

## Materials and methods

### Data sources

Healthcare professionals, marketing authorization holders, patients, and consumers voluntarily send AE reports to the PMDA. Information from the JADER database was obtained from the PMDA website (www.pmda.go.jp). All data from the JADER database were fully anonymized by the regulatory authority before we accessed them. The database consists of four data tables: patient demographic information, such as sex, age, and reporting year (demo); drug information, such as the non-proprietary name of the prescribed drug, route, and start and end dates of administration (drug); AEs, including the type, outcome, and date of onset (reac); and primary disease (hist). The evaluation period for this study was from April 2004 to March 2020. The “drug” file included the role codes assigned to each drug: suspected, concomitant, and interacting drugs. The suspected drug records were extracted and analyzed in this study.

### Definition of AEs

The AEs in the JADER were defined based on the Medical Dictionary for Regulatory Activities (MedDRA; www.meddra.org/how-to-use/support-documentation/japanese) version 19.0. For the extraction of cases from the JADER database, the preferred term (PT) peripheral neuropathy was used (PT code: 10029331).

### Drug selection

“Antineoplastic and immunomodulating agents” (ATC code: L) were evaluated according to the ATC classification system described by the World Health Organization Collaborating Centre for Drug Statistics Methodology (www.whocc.no/atc_ddd_index). The following nine ATC categories were related to CIPN with at least 10 reports: purine analogues (ATC code: L01BB), pyrimidine analogues (ATC code: L01BC), vinca alkaloids and analogues (ATC code: L01CA), taxanes (ATC code: L01CD), platinum compounds (ATC code: L01XA), monoclonal antibodies (ATC code: L01XC), protein kinase inhibitors (ATC code: L01XE), other antineoplastic agents (ATC code: L01XX), and other immunosuppressants (ATC code: L04AX). Epothilone (ixabepilone), listed as an antitumor drug related to CIPN, is not approved by the Japanese Pharmaceutical and Medical Devices Act (www.mhlw.go.jp/topics/bukyoku/hoken/iryokiki/dL/kokunai_list.pdf).

### ROR

**Figure 5:**
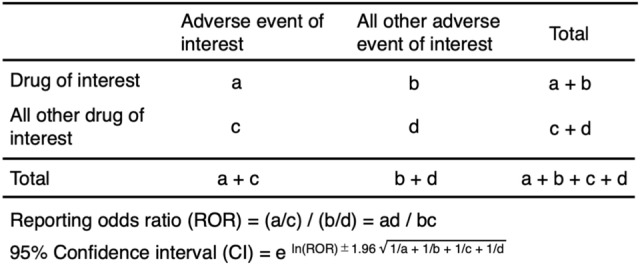
Two-by-two contingency table.

Signal detection for CIPN was based on the ROR that is commonly used in pharmacovigilance research^17^. The ROR is the ratio of the odds of reporting an AE relative to all other events associated with the drug of interest compared with the reporting odds for all other drugs in the JADER database. Each ROR was calculated using a two-by-two contingency table (Fig. 5). Signals were detected when the estimated ROR and lower limit of the corresponding 95% CI were greater than 1, and at least two cases were required to define the signal^18^.

### Time-to-onset analysis

The JADER database includes the date of the first administration of each drug and date of onset for each AE. Using data from the JADER database, the time between the initial date of drug administration and first occurrence of the AE was determined. The time to a specific AE from the prescription of specific drugs was evaluated using the median time, quartiles, and WSPs^19^. Duplicate prescriptions were excluded from the analysis. Additionally, reports that did not have complete AE occurrence or prescription start times were excluded. An analysis period of 365 days after the start date of administration was chosen. The scale parameter α determines the scale of the distribution function and shape parameter β determines the shape of the distribution function. A larger scale value (α) indicates a wider data distribution, whereas a smaller scale value shrinks data distribution. The shape parameter β of the Weibull distribution indicates the hazard without a reference population. When β is equal to 1, the hazard is estimated to be constant over time (random failure type). If the lower limit of the 95% CI of β is greater than 1, the hazard is considered to increase over time (wear-out failure type). If the upper limit of the 95% CI of β is less than 1, the hazard is considered to decrease over time (initial failure type)^19,46,47^.

The time-to-onset profiles of CIPN by sb-PTX and nab-PTX were compared between the groups using the Kaplan–Meier method with the log-rank test. Results with a *P* value of < 0.05 were considered statistically significant.

### Outcomes

To visually evaluate the relationship between the two types of categorical data, CIPN-related drugs (X) and outcomes (Y), a mosaic plot was constructed. Outcomes were classified as “death,” “with sequelae,” “not recovered,” “improved,” “recovered,” and “unknown.” Outcomes classified as “unknown” or blank were excluded.

All data analyses were performed using JMP Pro 16.0 (SAS Institute Inc., Cary, NC, USA).

### Ethical approval

Ethical approval was not sought for this study because the study was a database-related observational study without directly involving any research subjects. All results were obtained from data openly available online from the PMDA website (www.pmda.go.jp). All data from the JADER database were fully anonymized by the relevant regulatory authority before we accessed them.
