# Author Correction: Analysis of chemotherapy-induced peripheral neuropathy using the Japanese Adverse Drug Event Report database

**DOI:** 10.1038/s41598-022-24702-8

**Published:** 2022-11-28

**Authors:** Misaki Inoue, Kiyoka Matsumoto, Mizuki Tanaka, Yu Yoshida, Riko Satake, Fumiya Goto, Kazuyo Shimada, Ririka Mukai, Shiori Hasegawa, Takaaki Suzuki, Hiroaki Ikesue, Jun Liao, Tohru Hashida, Mitsuhiro Nakamura

**Affiliations:** 1 Laboratory of Drug Informatics, Gifu Pharmaceutical University, Gifu‑shi, Gifu Japan; 2 Department of Pharmacy, Kobe City Medical Center General Hospital, Kobe‑shi, Hyogo Japan; 3 Gifu Prefectural Government, Gifu‑shi, Gifu Japan; 4 Department of Pharmaceutical Informatics and Biological Statistics, School of Science, China Pharmaceutical University, Nanjing, China

Correction to: *Scientific Reports* 10.1038/s41598-021-90848-6, published online 31 May 2021

The original version of this Article contained an error in Figure 3, where the red line representing solvent-based (sb)-paclitaxel was incorrectly captioned as “nanoparticle albumin-bound (nab)-paclitaxel”, and the blue line representing nanoparticle albumin-bound (nab)-paclitaxel was incorrectly captioned as “solvent-based (sb)-paclitaxel”.

The original Figure 3 and accompanying legend appear below.

**Figure 3:**
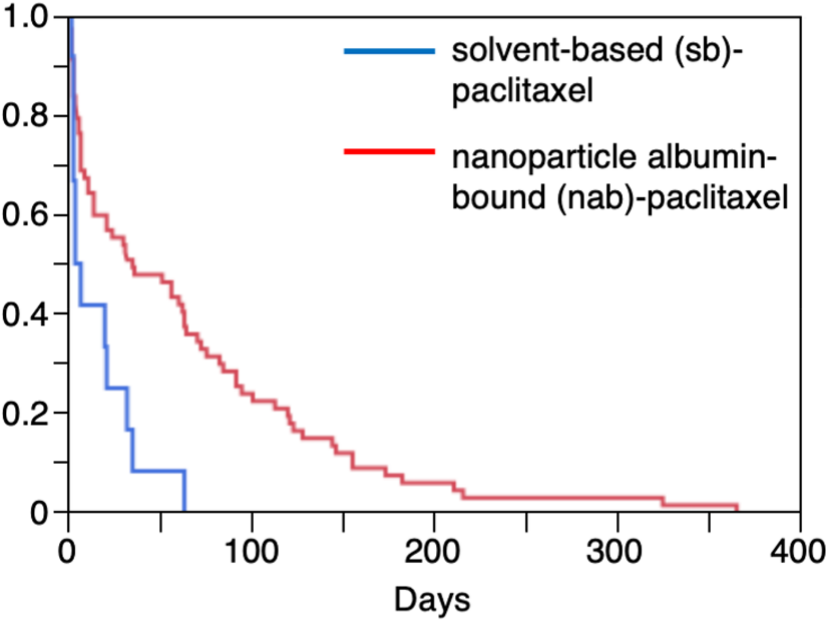
Kaplan–Meier plot of chemotherapy-induced peripheral neuropathy for solvent-based (sb)-paclitaxel and nanoparticle albumin-bound (nab)-paclitaxel.

The original Article has been corrected.

